# Genetic colour variation visible for predators and conspecifics is concealed from humans in a polymorphic moth

**DOI:** 10.1111/jeb.13994

**Published:** 2022-03-03

**Authors:** Ossi Nokelainen, Juan A. Galarza, Jimi Kirvesoja, Kaisa Suisto, Johanna Mappes

**Affiliations:** ^1^ Department of Biological and Environmental Science University of Jyväskylä Jyväskylä Finland; ^2^ Organismal and Evolutionary Biology Research Program Faculty of Biological and Environmental Sciences University of Helsinki Helsinki University Helsinki Finland

**Keywords:** aposematism, *Arctia plantaginis*, discriminant analysis, multispectral imaging, polymorphism, wood tiger moth

## Abstract

The definition of colour polymorphism is intuitive: genetic variants express discretely coloured phenotypes. This classification is, however, elusive as humans form subjective categories or ignore differences that cannot be seen by human eyes. We demonstrate an example of a ‘cryptic morph’ in a polymorphic wood tiger moth (*Arctia plantaginis*), a phenomenon that may be common among well‐studied species. We used pedigree data from nearly 20,000 individuals to infer the inheritance of hindwing colouration. The evidence supports a single Mendelian locus with two alleles in males: WW and Wy produce the white and yy the yellow hindwing colour. The inheritance could not be resolved in females as their hindwing colour varies continuously with no clear link with male genotypes. Next, we investigated if the male genotype can be predicted from their phenotype by machine learning algorithms and by human observers. Linear discriminant analysis grouped male genotypes with 97% accuracy, whereas humans could only group the yy genotype. Using vision modelling, we also tested whether the genotypes have differential discriminability to humans, moth conspecifics and their bird predators. The human perception was poor separating the genotypes, but avian and moth vision models with ultraviolet sensitivity could separate white WW and Wy males. We emphasize the importance of objective methodology when studying colour polymorphism. Our findings indicate that by‐eye categorization methods may be problematic, because humans fail to see differences that can be visible for relevant receivers. Ultimately, receivers equipped with different perception than ours may impose selection to morphs hidden from human sight.

## Graphical abstract

The definition of colour polymorphism is intuitive: genetic variants express discretely‐coloured phenotypes. We show that genetic colour variation visible for predators and conspecifics is concealed from humans in the polymorphic wood tiger moth. Here, human visible colour variation (a white male, a yellow male and an orange female from top to bottom) is depicted.

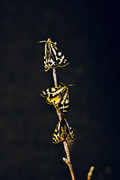

## 1 INTRODUCTION

Colour polymorphism, the occurrence of multiple discrete colour phenotypes within a population (Ford, 1945; Huxley, 1955; White & Kemp, 2016), is a flagship topic of evolutionary biology (Gray & McKinnon, 2007; McKinnon & Pierotti, 2010; Svensson, 2017). The study of colour polymorphism has traditionally been a very popular topic among evolutionary biologists (Brakefield & Liebert, 1985; Cain & Sheppard, 1954; Fisher & Ford, 1947; Kettlewell, 1955), because as a visible trait, colouration enables scientists to study evolution in action in a tractable manner. Importantly, colouration is a composite trait that has multiple fitness‐linked functions (Cuthill et al., 2017), including thermoregulation (e.g. Stuart‐Fox et al., 2017), immune defence (e.g. Freitak et al., 2005), sexual signalling (e.g. Tibbetts et al., 2017) and avoiding predation either through camouflage, mimicry or warning signalling (e.g. Ruxton et al., 2019). The diversity of colours, as well as colour polymorphism, is therefore valuable to understand the processes generating and maintaining genetic variation in the wild.

Classically, a genetic polymorphism is defined as: ‘the occurrence together of two or more discontinuous forms of a species in the same habitat in such proportions that the rarest of them cannot be maintained merely by recurrent mutation’ (Ford, 1945, 1965; Huxley, 1955). This definition has remained virtually unchanged over the last 75 years (Nokelainen et al., 2018; Svensson, 2017; White & Kemp, 2016). While the concept of colour polymorphism may be rather intuitive, its quantification is not, mainly because the difference between colour variants is not always clear‐cut. For example, phenotypic plasticity can provide discrete appearances as if they were a result of polymorphism (Price, 2006), such as the density‐dependent colour change (i.e. polyphenism) in a desert locust (*Schistocerca gregaria*) (Sword et al., 2000). On the other hand, sometimes a genetically polymorphic trait may show overlapping phenotypic distribution (Kappers et al., 2018; Nokelainen et al., 2018), such as the extravagant colour polymorphism of the Hawaiian happy‐face spider (*Theridion grallator*) that shows high phenotypic variation among populations (Gillespie & Oxford, 2009).

In Lepidoptera, one of the potential caveats of early polymorphism studies is that colouration was mostly quantified through human vision and thus included a source of subjectivity (Brakefield & Liebert, 1985; Endler, 1990; Fisher & Ford, 1947). This may not always be a problem as humans are good in categorizing colours across a broad visible wavelength spectrum from 400 to 700 nm (Bergeron & Fuller, 2017) and have excellent visual acuity (Caves et al., 2018). However, our perception excludes the near ultraviolet part of the spectrum (300–400 nm), which is important to many animals in signalling (Kelber et al., 2003; Osorio & Vorobyev, 2005). Also, humans may not be able to detect nuances in patterns or judge colour polymorphism based only on a single key trait (e.g. hindwing ‘base’ colour), or see subjective categories where they do not exist. Colouration must therefore be objectively quantified using either spectrometry or multispectral imaging approaches (Endler, 1990, Troscianko & Stevens, 2015, van den Berg et al., 2020). As such there is a clear need for studies that can link the phenotypes to their genotypes, because this can illuminate our understanding of how selection of allele frequencies that constitute the genotypes operate in the wild (Cuthill et al., 2017; Svensson, 2017; Tibbetts et al., 2017).

We investigated genotype–phenotype associations in the wood tiger moth (*Arctia plantaginis*), a widely distributed member of the Erebidae family (Rönkä et al., 2016) found across the Northern hemisphere (Hegna et al., 2015). It is known that its polymorphic hindwing colour is heritable in males (Nokelainen et al., 2013) and in females (Lindstedt et al., 2016). In general, males have either yellow or white hindwings (Nokelainen et al., 2012, 2013; Suomalainen, 1938). Female hindwings, on the other hand, vary continuously in the yellow‐orange‐red range (Lindstedt et al., 2011).

First, we explored the heritability of the hindwing colouration. It has been suggested, although with a very limited data set originating from a single brood, that the inheritance of male hindwing colouration follows a Mendelian one‐locus two‐allele model where the yellow allele is recessive (Suomalainen, 1938). The genetic mechanism of female wing colouration is largely unknown and some plasticity in female colouration has been reported (Lindstedt et al., 2010). To confirm the mode of inheritance, we compared human‐visible hindwing colour (and only hindwing colour as genotype proxy) frequencies from 452 laboratory‐reared families (i.e. with pedigree) against those predicted by the one‐locus two‐allele model. Second, focussing in the males, we tested further whether their genotype can be predicted by their phenotype. We examined if the colour morphs could be assigned to their genotype (i.e. an information derived from the pedigree, Box 1) by human observers through sequential and simultaneous sorting tasks, as well as by machine learning algorithms (i.e. discriminant functions). We used linear discriminant function analysis as part of the machine learning realm, as we wanted to explicitly understand what are the parameters that may allow visual separation of the genotypes. It can be expected that the computational methods should outperform human sorting skills, because the algorithms can take into account combined nuances in phenotypic variation, including those beyond the human‐visible spectrum (Høye et al., 2021; Wilkins & Osorio, 2019). Lastly, we asked whether these genotype–phenotype associations may have ecological relevance beyond the human‐visible spectrum. Using vision modelling, Henze et al. (2018) investigated the differences in the discriminability of the wood tiger moth colour morphs by moth conspecifics and bird predators. Here, we used receptor‐noise‐limited vision modelling (Maia et al., 2013; Vorobyev & Osorio, 1998) to test pairwise genotype chromatic contrasts of hindwing colour using human, avian and moth vision models.

BOX 1 **Pedigree crossing design and determination of the wood tiger moth genotypes with respect to hind wing colour. The first panel shows the classic Mendelian one locus two allele segregation (A); we expect that white is dominant trait over yellow (Suomalainen, 1938). Each homozygous parent in the parental generation produces one type of gamete (W or y). The following generation heterozygous offspring produces again two types of gametes. In the second panel (B), the next generation produces offspring with a 3:1 ratio of dominant allele to recessive. The third panel, shows the crossing design followed to mate selection lines of known genotypes and their expected phenotype frequencies. Fifteen generations were produced over the course of 6‐years. The colours in the bars indicate the hind wing colour of the offspring. An important point with this classic approach is that by producing consecutive generations and following the logic of expected offspring phenotype frequencies, it is possible to back‐trace pedigree and determine putative genotypes of earlier generations.**

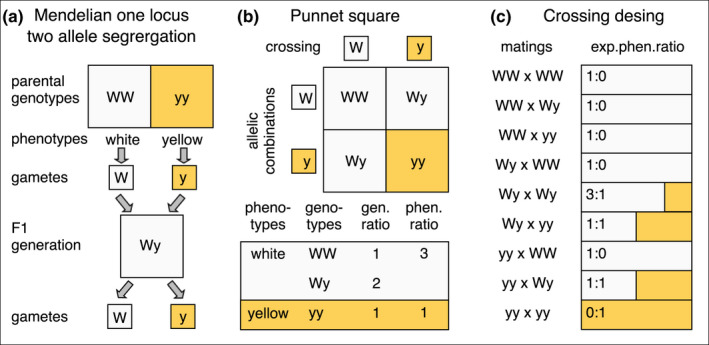

## 2 MATERIAL AND METHODS

### 2.1 Moth pedigree rearing protocol

**FIGURE 1:**
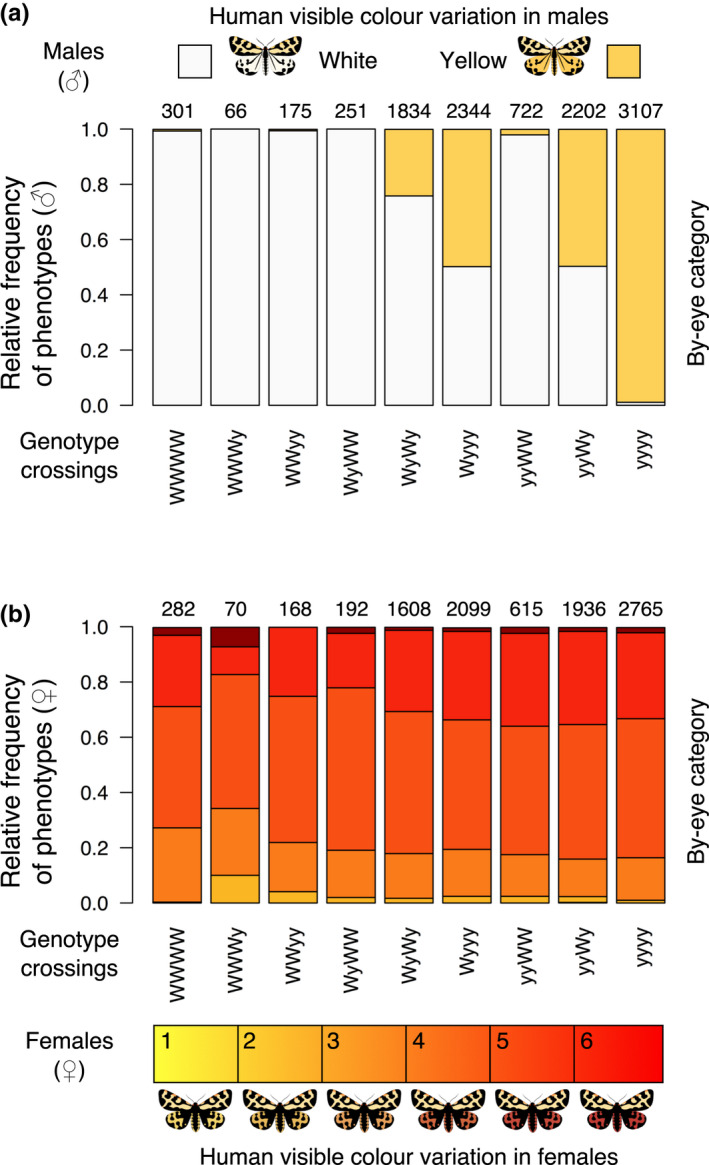
Pedigree information of the wood tiger moth genotype crossings and their human‐visible hindwing coloration. The figure shows relative frequencies of offspring phenotypes with respect to their parental genotype crosses. The numbers above the bars indicate the sample size. The colours in the bars indicate the subjective by‐eye hindwing colour of the offspring. Notice the dichotomous yellow‐white hindwing categorization in males (a), whereas the females are more converged to similar orange coloration (b); the scale depicts the visually scaled yellow‐orange‐red colour gradient used to categorize female coloration. The images were gamma corrected for better screen imaging and are meant to illustrate representative examples of the wood tiger moth colour variation. In males, hindwing colour shows statistically nonsignificant difference from predicted one locus two allele model, whereas females deviate significantly from the same predicted outcome of phenotypes

Altogether we used pedigree data from 15 generations of the wood tiger moth reared in the laboratory over the course of 6‐years. As a general rearing protocol, two adults (male and female) are put together in a plastic box (Huhtamäki, 1000 ml, transparent casing) with mesh for ventilation at the top and allowed to mate under natural lighting. The eggs are laid inside the box where after ~6 days the larvae hatch and are kept for another ~14 days inside the box as they are too delicate to be moved. The larvae are then separated into rearing containers (max. 30 larvae/container) to continue growth at approximately 25°C under natural light conditions and are fed with fresh dandelion leaves (*Taraxacum ssp*.) until pupation. The pupae are then moved into individual jars and sex and wing colour are recorded from the emerging adults. In males, colour classification is conventionally done by‐eye (white or yellow hindwing ‘base colour’) and in females, a six‐step (yellow‐orange‐red) scale is used as described by Lindstedt et al. (2010) and Nokelainen et al. (2012): in 1 to 6 scale yellow‐orange‐red gradient yellows are 1–2, oranges 3–4 and reds 5–6 (Figure 1).

### 2.2 Inheritance of hindwing colour based on the pedigree

We specifically tested a Mendelian inheritance model where a single locus with two‐alleles controls the white‐yellow polymorphism. Here, the yellow allele (y) is recessive to the dominant white (W) allele, as suggested by Suomalainen (1938) who used a single brood of individuals originating from Finland. Under this model, genotypes with the W allele (i.e. WW, Wy) exhibit white hindwings, whereas only the homozygote recessive genotype (i.e. yy) exhibit yellow hindwings. We tested the one‐locus two‐allele model (Box 1), by comparing the expected model's frequencies to those observed from 452 families with pedigree data (each offspring was considered as independent data point) using a Chi Square test for independence. The expected frequencies under this model with their resulting hindwing colour are presented in Tables S1 and S2. Only families with at least 10 male and female offspring were used to ensure reliable frequency distribution. From the pedigree data, the parental genotypes were inferred from the phenotype distribution of the F1 offspring. For instance, a 100% yellow male offspring would indicate that both parents are homozygous for the y allele (Box 1). The reasoning uses the same approach as myriad classic studies of ecological genetics deciphering Mendelian ratios. The key point with this pedigree back‐trace approach is that by producing consecutive generations it is possible to match the observed F1 phenotype frequencies to those expected by Mendelian inheritance, and thus, the genotype of the parental generation can be inferred.

### 2.3 Objective genotype–phenotype associations using image analysis techniques

The pedigree back‐trace approach above was used to unravel the inheritance of hindwing colouration using hindwing colouration as perceived by humans. To investigate genotype–phenotype associations further than simply using a human‐visible hindwing colour, we used a subset of laboratory‐reared adults with a known pedigree in our image analyses. The total sample size of the photographed individuals was 292: where of the males 37 were WW, 88 were Wy, and 42 were yy, while of the females, 33 were WW, 68 were Wy and 24 were yy. The image calibration and analysis broadly followed previously established methods (Troscianko & Stevens, 2015, van den Berg et al., 2020). Briefly, photography was undertaken with a Samsung NX1000 digital camera converted to full spectrum with no quartz filter to enable UV sensitivity fitted with a Nikon EL 80 mm lens. For the photos in the human‐visible range, we used a UV and infrared (IR) blocking filter on the lens, which passes wavelengths only between 400 and 680 nm (Baader UV/IR Cut Filter). For the UV images, a UV pass filter was used (Baader U filter), which transmits wavelengths between 320 and 380 nm. Grey reflectance standards, which reflect light equally at 7% and 93% between 300 and 750 nm, were used for image calibration. A standard light source 75W Exo‐terra Sunray (mimicking sunlight across the spectrum) was used.

To obtain colour and pattern metrics, we measured the entire dorsal view of the forewings (FW), hindwings (HW), thorax (TH) and abdomen (AB) of the mounted and spread adult as regions of interest (ROI). For reflectance data, we used normalized camera responses of red, green, blue and the UV channel. To extract pattern information, we applied a pattern analysis technique (a ‘granularity’ analysis), which decomposed the image into a series of spatial frequencies (‘granularity bands’) using Fourier analysis and band pass filtering, followed by determining the relative contribution of different marking sizes to the overall pattern (Barbosa et al., 2008; Hanlon et al., 2009; Stoddard & Stevens, 2010). The analysis calculated the amount of light information (or pixel energy) corresponding to markings of different sizes, starting with small markings (we used a pixel start size of 2) and increased in size to larger markings (we used a pixel end size of 100). Increase in pixel step size was set to multiply each step by 1.414, thus representing exponential growth. The luminance was measured over 20 bands from lowest luminance (0) to highest luminance (65535), the maximum dynamic range of a 32‐bit TIFF image. The luminance channel was set to longwave channel (R). For the pattern data variables, we used dominance (i.e. maxPower—the energy at the spatial frequency with the highest pixel energy), diversity (i.e. propPower—maximum or peak energy value divided by the summed energy) and marking size (i.e. maxFreq—the spatial frequency with peak energy).

Prior to testing, colour metrics were filtered for correlations to avoid multicollinearity. The following variables were retained: area, three pattern variables (pattern size, contrast and diversity) and four bandpass channels (UV, blue, green, red channels, i.e. uv, sw, mw, lw respectively). All values were separately measured for the four regions of interest (forewing, hindwing, thorax and abdomen). In addition, the following allometric measurements of size were calculated by dividing areas of the ROIs: forewing to abdomen (FW/AB), forewing to thorax (FW/TH), forewing to hindwing (FW/AB) and thorax to abdomen (TH/AB).

### 2.4 Discreteness of colour morphs—subjective genotype discrimination using human observers

Next, we evaluated human sorting accuracy of male genotypes through sequential (‘sequence’) and simultaneous (‘sorting’) tasks. Participants were familiar with the wood tiger moth. In both tasks, we showed participants 10 images per genotype (i.e. 3 genotypes by 10 replicates) and asked them to sort the images according to their genotype. As the mechanism controlling for the hindwing colouration in females is currently unknown and warrants further investigation, we used only males in the sorting tasks due to their discrete hindwing colouration that allows for tractable inheritance.

In the sequential sorting task, 12 participants were asked to classify moth photographs by their genotype. The photographs were of a mounted specimen with wings spread out. The photographs were shown in a randomized order via the Google Docs ‘Forms’ platform. The participants were asked to pay attention to the appearance, wing and body colouration. The following cues derived from the image analysis (see above) to classify the male genotypes (WW, Wy, yy) were given as training instructions. White hindwings, large forewing patterning and pale abdomen are typical to WW. White hindwings and a yellow tinge in forewings and abdomen are typical to Wy. Yellow hindwings, variable wing patterning, yellow abdominal colour is typical to yy. The participants were instructed to classify moths using these cues (Fig. S3).

In the simultaneous sorting task, 10 participants (a subset of the former group) were asked to sort the genotypes into three clusters based on similarities in their appearance; no further instructions were given to accomplish this task. All moths were visible at the same time and the test was done using PowerPoint slides with moth photographs (i.e. 30 images were simultaneously presented, 3 genotypes by 10 replicates). The percentage of correct answers was then calculated (Fig. S4).

### 2.5 Vision modelling

The vision modelling we carried out largely followed established methods (Stevens et al., 2007, Troscianko & Stevens, 2015, van den Berg et al., 2020). To gain insight into how well different vision systems can recognize the colour differences between genotypes, we used a receptor‐noise limited (RNL) visual discrimination model (Vorobyev et al., 1998). We compared trichromatic human, tetrachromatic avian (Blue tit; *Cyanistes caeruleus*) and trichromatic moth (wood tiger moth) vision models. This allowed us to mechanistically understand human sorting accuracy of genotypes and to compare this with more ecologically relevant vision systems of conspecifics (moths) and predators (birds). We used 0.05 Weber fraction for most abundant cone type for all vision models. The cone ratios were: avian cone ratios 1:1.92:2.68:2.7 uv:sw:mw:lw (Hart, 2001b), human cone ratios 0.057:0.314:0.629 sw:mw:lw (Hofer et al., 2005). For moth vision model, spectral sensitivities of cone cells (uv, sw, mw) were obtained from (Henze et al., 2018), and cone ratios 1:1:1, were used as the specific ratio is unknown. As we were interested in differences in chromatic contrast (dS), we excluded the achromatic contrast (dL) from the vision model analysis. The vision model yields discrimination values in ‘just noticeable differences’ (JNDs), although before behavioural validation these should be considered as predicted contrast values (dS). By definition, values lower than one (<1 JND) are considered indistinguishable, whereas larger values are discriminable for the receiver (Kang et al., 2015; Nokelainen et al., 2019; Siddiqi et al., 2004).

### 2.6 Statistical analyses

First, we tested whether the wood tiger moth hindwing colour follows a simple Mendelian one‐locus two‐allele inheritance pattern using Chi Square test for independence. We would expect that the observed phenotype frequencies from the crossing designs do not deviate significantly from the predicted phenotype frequencies. We tested the expected versus observed subjective colour morph frequencies separately for males (Table S1) and females (Table S2).

Second, we tested the discriminability of genotype–phenotype associations. The success rate of correct genotype designation was tested with a general linear model (GLM with a Poisson distribution), where the success rate of visually genotyping each moth was set as the dependent variable and method of genotyping (human sequential task, human simultaneous task, or computer algorithm) as the explanatory variable. We used a linear discriminant function analysis to evaluate whether the computer algorithm can outperform human observers in the genotype sorting task. The analysis was carried out as a 3‐group problem. The genotype (derived from the pedigree data) was set as the predicted group membership and regions of interest (ROI) were selected form the digital image namely; area, pattern size, pattern contrast, pattern diversity, uv, sw, mw, lw, forewing to abdomen, forewing to thorax, forewing to hindwing and thorax to abdomen were set as predictor variables. All colour and pattern metrics were investigated separately for the forewing, hindwing, thorax and abdomen. We also used the Boruta feature selection algorithm to provide additional information on which features are the best predictors of the prior genotype groups (under R‐package ‘Boruta’). Briefly, Boruta is a random forest algorithm that compares the significance of each variable against random noise data created from all variables of interest (Kursa & Rudnicki, 2010). The significance is then determined based on the relative difference against the random noise. Generally, variables, which fall in between lower‐ and upper‐bound significance of the random generated noise reference are flagged as non‐significant (Table S3, Figs. S1–S2).

Third, we tested the discriminability of the genotypes using three different vision models. For this, we conducted a linear mixed effects model (lmer‐function) with the lmerTest R‐package (Kuznetsova et al., 2017). The colour contrast (dS) was set as the dependent variable and genotype (WW, Wy, yy), ROI (abdomen, forewing, hindwing), vision model (avian, human, moth) and their interactions were set as the explanatory variables. The moth ID was set as a random variable to control for data structure. All analyses were conducted using RStudio, version 1.1.447 and R, version 3.5.0 (R Core Team, 2018; RStudio Team, 2016).

## 3 RESULTS

### 3.1 Mendelian inheritance of the hindwing colour polymorphism

Of the individuals used in the pedigree analysis 10911 were males and 8295 were females (Figure 1, Tables S1 and S2). In the males, the frequencies of white and yellow offspring were in close agreement to the expected phenotype frequencies under one‐locus two‐allele Mendelian inheritance where white dominates over yellow (Box 1, Figure 1a). Thus, the one‐locus two‐allele inheritance mode with dominance of the W allele over y was confirmed by the pedigree data for males. Whether the established locus also controls hindwing colour in the females was less clear. In females, the spread of the hindwing colour phenotypes showed an apparent normal distribution (Figure 1b) and indicated phenotypic tendency towards orange hindwing colouration (by‐eye classification; yellow‐orange‐red). Out of all crossings, the emerged females on average, were 3% yellow, 68% orange and 29% red regardless of the parental genotype. Thus, there was no obvious correlation between the male (white‐yellow) and female (yellow‐orange‐red) hindwing colour within the brood (Figure 1a‐b) when using the subjective hindwing colour classification made by human observers.

### 3.2 Discriminability of genotype–phenotype associations—human versus algorithm

**FIGURE 2:**
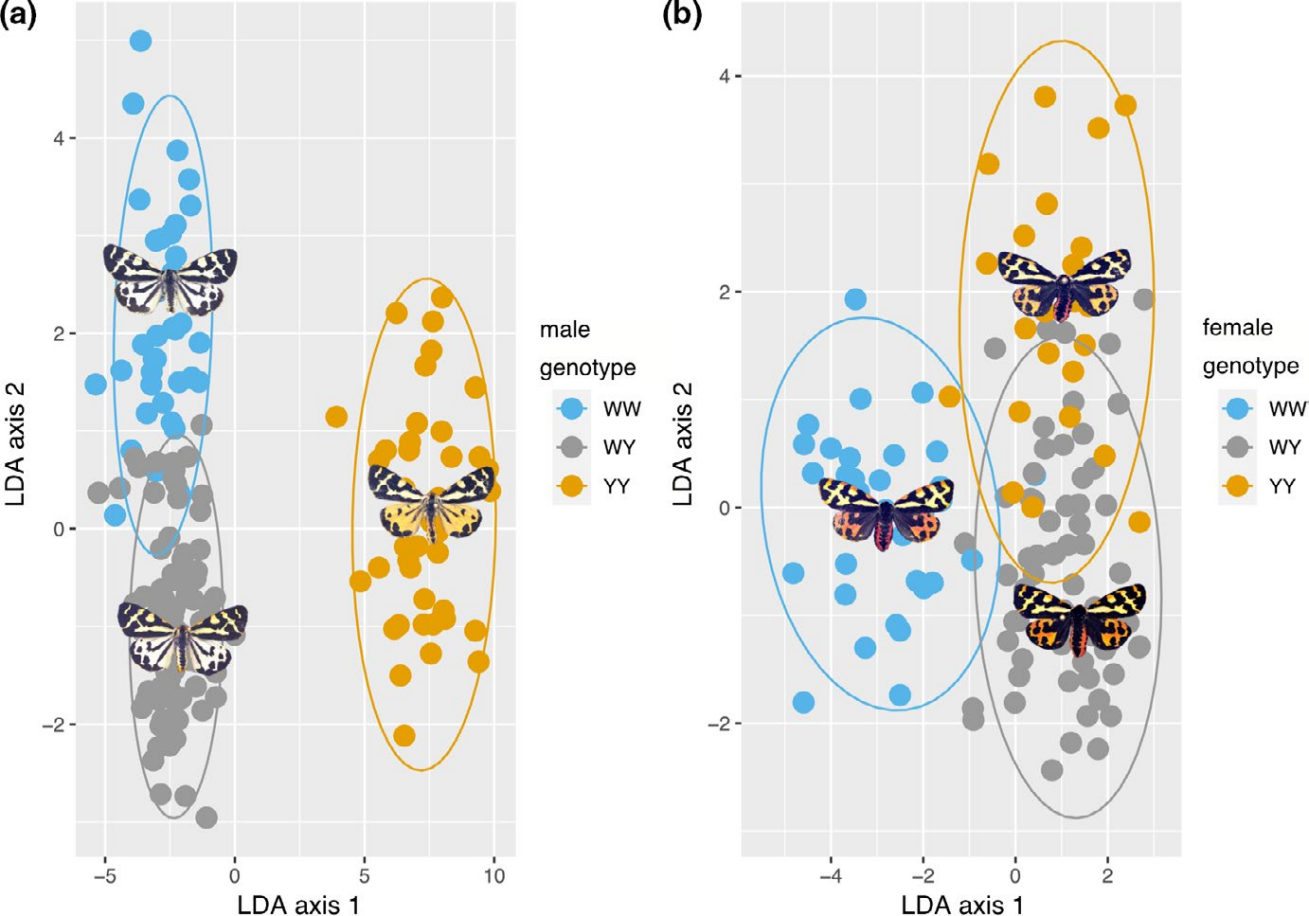
Objective phenotype quantification using colour and pattern metrics. Linear discriminant analysis for wood tiger moth male (a) and female (b) genotypes. Notice that here genotypes refer to one locus two allele model, where there are two predominantly white morphs and one yellow in males and red, orange, yellow morphs in females. In males, the yy (yellow) genotype is clustered easily as its own subgroup, but linear combinations also separates WW (white) and Wy (white) genotypes with good degree of certainty along a second axis that combines parts of the full spectrum (invisible to us) as well as pattern metrics. In females, the three genotypes seemingly cluster into three subgroups; however, there is much more grouping overlap (i.e. phenotypic convergence) than in males

A computer‐based discrimination algorithm outperformed subjective sorting accuracy of humans using linear combinations of colour and pattern data (ANOVA: *F*_2,48_ = 7.10, *p* < 0.001). In males, the linear discriminant analysis reached 96.80% accuracy for predicting the correct genotype membership. Within test data, 88.88% of WW, 98.11% of Wy and 100% of yy were correctly classified (Figure 2a). The discriminability of genotypes using colour metrics was also assessed using the Boruta feature extraction algorithm (Table S3). The most important variables to separate white and yellow morphs are all hindwing features and include: UV reflectance, short wavelength reflectance, pattern diversity, pattern contrast and long wavelength reflectance. The most important variables to separate white genotypes (WW, Wy) are: thorax UV reflectance, thorax to abdomen size ratio, abdomen UV, forewing to abdomen size ratio and forewing pattern diversity.

In females, the linear discriminant analysis reached 65.65% accuracy for predicting the correct genotype membership. Within test data, 83.33% of WW, 58.62% of Wy and 69.56% of yy were correctly classified (Figure 2b). The most important variables to make a distinction between white homozygotes (WW) and yellow allele bearers (Wy or yy) are: thorax UV reflectance, abdomen marking size, abdomen UV reflectance, forewing UV reflectance, hindwing short wavelength reflectance (Table S3). The most important variables to separate white heterozygotes (Wy) from yellow homozygotes (yy) are: abdomen marking size, hindwing short wavelengths, hindwing medium wavelengths, forewing short wavelengths and hindwing long wavelengths.

**FIGURE 3:**
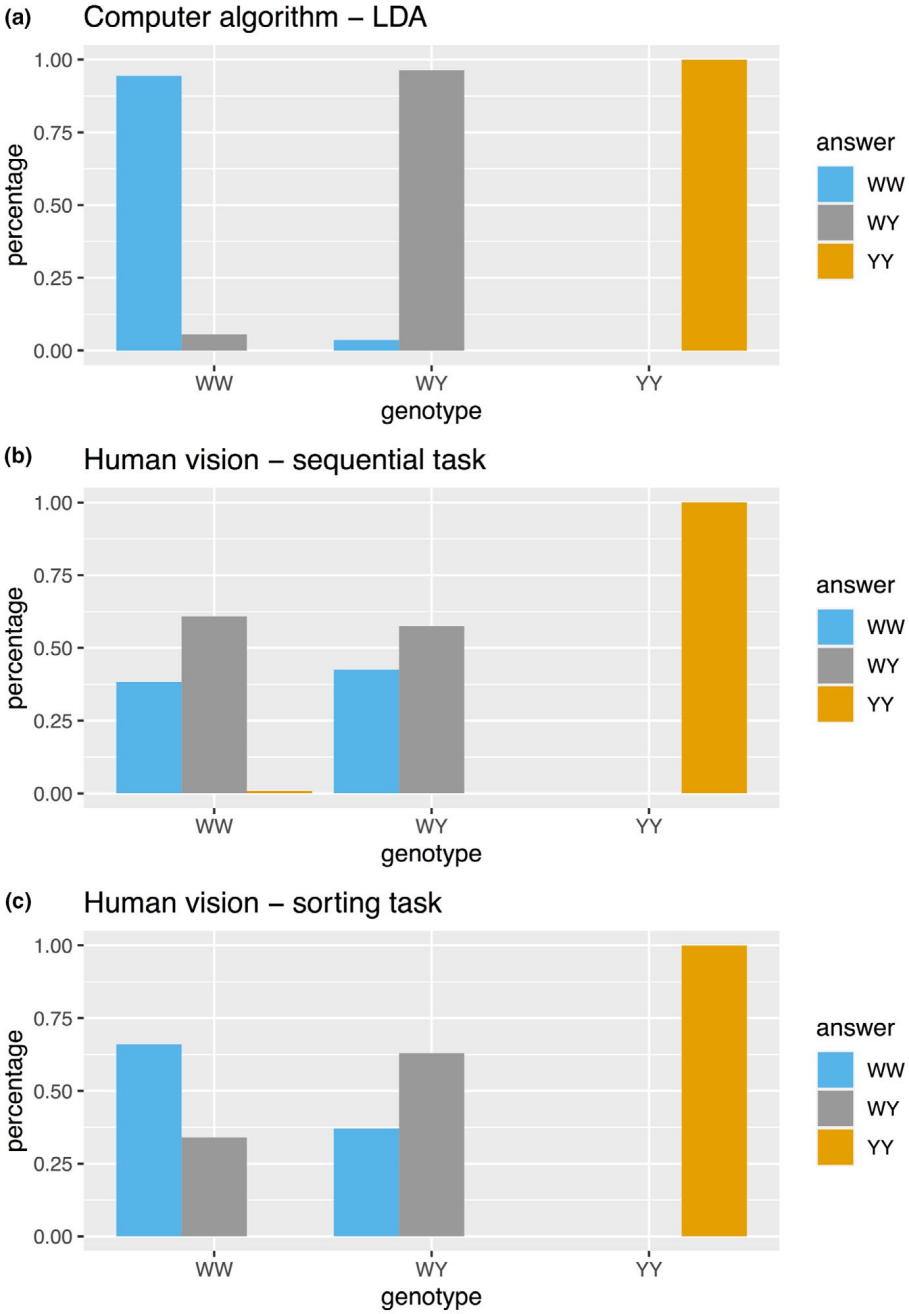
The success of objective versus subjective sorting of male genotypes. Colour metric supervised computer algorithm (LDA, linear discriminant analysis) outperforms human sorting accuracy of genotypes in both sequential (‘sequential’) and simultaneous (‘sorting’) tasks. In sequential sorting people were asked to classify moth pictures by their genotypes; only basic information of the best describing colour metrics were given as instructions. In simultaneous sorting, people were asked to sort the genotypes into three clusters based on their superficial appearance; no further instructions were given, but all moths were visible at the same time. Percentage indicates the number of correct answers by genotype

We next focussed on discriminability of genotypes for human observers only using males as we detected a close phenotypic similarity in females. Human participants were not able to reliably categorize the genotypes (Figure 3, Fig. S5). Of the male moths, participants were only able to distinguish yellow (yy) genotype from the whites, but not the two white male genotypes (WW, Wy). In the simultaneous sorting task where all moths were presented together, participants were able to sort the homozygous and heterozygous males only slightly better than in the sequential sorting task, yet only 60% was the highest success rate in sorting white males based on genotype.

### 3.3 Chromatic discriminability of the genotypes to ecologically relevant receivers

**FIGURE 4:**
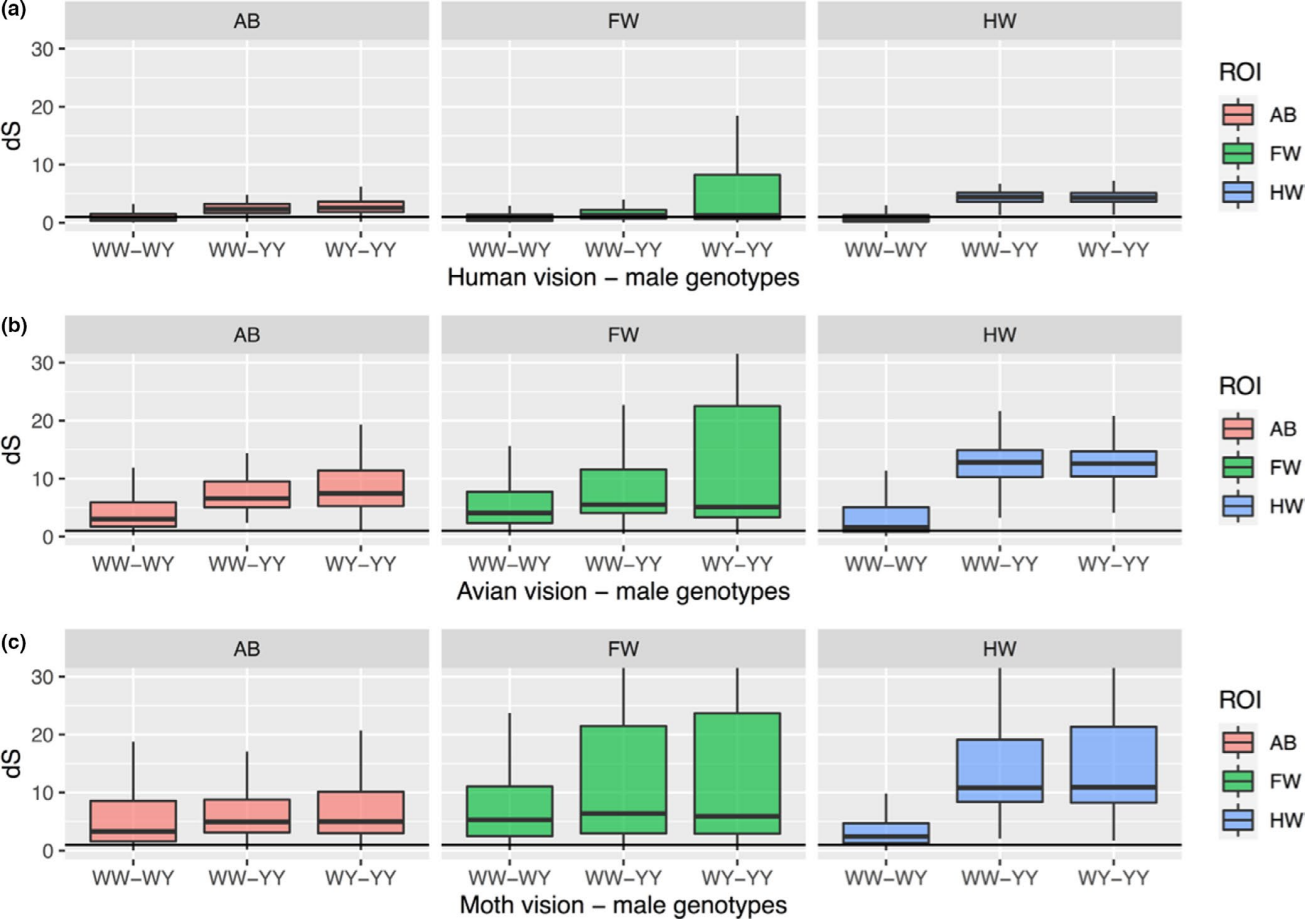
Wood tiger moth genotype separability through ecologically relevant vision systems compared to human vision. We tested the discriminability of the genotypes using three different vision models (human, avian and moth vision). In all images, x‐axis represents pairwise genotype comparisons and y‐axis shows chromatic contrast (dS) of the two moths being compared. The region of interest (ROI) indicates the comparison between abdomen (AB), forewing (FW) and hindwing (HW) colour. The panels separate vision modelling results for human (a), avian (b) and moth (c) vision models. Contrast values <1 are considered indistinguishable and values above this are increasingly easy to distinguish (outliers not shown). The black horizontal line indicates dS = 1 corresponding to the perception threshold of the contrast difference

**FIGURE 5:**
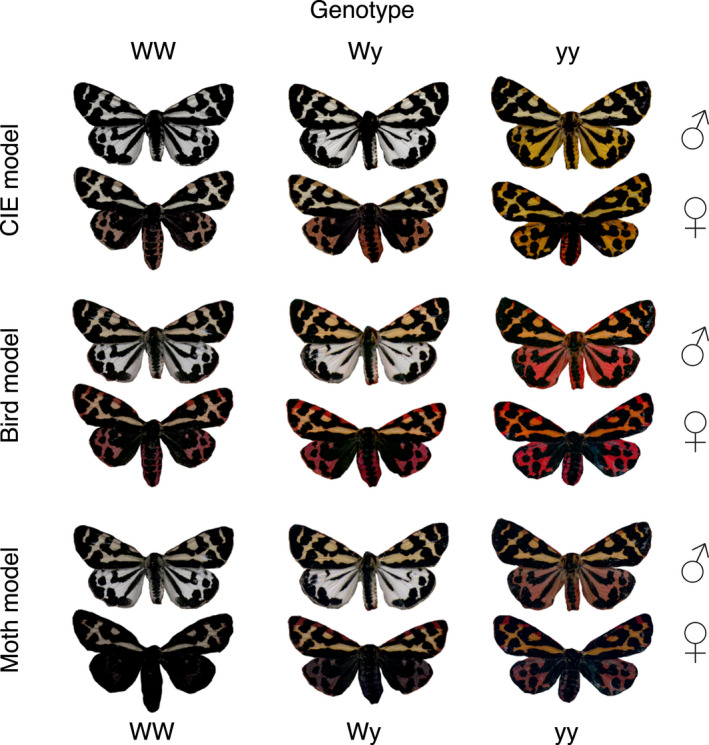
Representation of the wood tiger moth genotypes to illustrate how they may appear to ecologically relevant receivers. These false image examples show genotypes of both sexes organized in vertical columns and human, avian and moth false colour images in horizontal groups. For trichromatic human vision sw, mw and lw sensitivities were used for blue, green and red channels respectively. For tetrachromatic avian vision uv, sw and lw sensitivities were used for blue, green and red channels respectively. For trichromatic moth vision uv, sw and mw sensitivities were used for blue, green and red channels respectively. Images were corrrected for better image screening

The discriminability of the genotypes was measured pairwise using hindwing chromatic contrast (dS) of the two moths being compared (Figure 4). The vision modelling results indicate that detectability of the genotypes was different for human, bird and moth vision models, as the three‐way interaction of vision model, ROI and genotype was significant (lmer ANOVA, *F*_4,23028.1_ = 506.11, *p* < 0.001). Also, vision modelling results suggest that human perception is poor at separating WW and Wy male genotypes, whereas avian and moth vision systems with UV sensitivity could separate white WW and Wy male genotypes (Figure 4). Thus, when viewed through ecologically relevant receivers’ vision, the genotypes may have nuanced phenotypic differences beyond human perception (Figure 5).

## 4 DISCUSSION

Our results highlight that genetic polymorphism expressed at the phenotypic level is not always clear‐cut to define as categorization depends on the perception. With a wood tiger moth stock originating from north Europe, we validate that male hindwing colour is genetically controlled by a single Mendelian locus, where the white allele (W) is dominant to the yellow (y) allele. Male genotypes can be told apart using colour metrics with high accuracy by machine learning algorithms, but not by human observers, because white heterozygous males can be separated from white homozygotes by differences in ultraviolet reflectance. In turn, female genotypes are currently inseparable by their phenotypes to us. It seems plausible that female colour is controlled to some extent by the same genetic loci as the male colour, although with different dominance relationships and other interacting loci. Although we have less‐extensive data about colour variation in females, it seems that in many localities a yellow‐orange‐red continuum is common (Lindstedt et al., 2011).

Generally, functional genes in the melanin biosynthetic pathway can affect both wing scale pigmentation and morphology in Lepidoptera (Matsuoka & Monteiro, 2018). Our preliminary pigment analyses in the wood tiger moth indicate that the white pigmentation in the wings is produced by N‐acetyldopamine (NADA) sclerotin, whereas the yellow pigment is derived from a mix of N‐β‐alanyldopamine (NBAD) sclerotin and pheomelanin and the red pigment results from a dopamine‐derived pheomelanin (Brien et al. In Prep.). Also, differential expression in melanin‐promoting and melanin‐inhibiting genes impacts black colouration in the cuticle and in the hairs of wood tiger moth caterpillars (Galarza, 2021). Plausibly, differential regulation in genes involved in the melanin pathway could contribute to the colour differences between the sexes (Gazda et al., 2020). Whether an upregulation of a single gene that causes pigment degradation in the other sex could take place in wood tiger moth's melanin‐based colouration is currently unknown but warrants further investigation.

The machine learning algorithm was more efficient at assigning individuals to known group memberships based on the subtle, but consistent phenotypic differences between the genotypes in comparison to human observers. The finding that computational approach outperforms human perception is not surprising, but still the majority of the colour polymorphism research relies on classic approach using human‐visible categorization. In males, the linear discriminant analysis separated the three‐group problem with high accuracy. Successful discrimination between the three groups of males was expected due to differences in short and long wavelength reflectance between the two white morphs (Henze et al., 2018; Nokelainen et al., 2012). Also, the white homozygotes have a lower thorax UV reflectance, smaller thorax by abdomen ratio (i.e. larger abdomens), smaller forewing by abdomen ratio, lower abdominal UV reflectance and less variable forewing patterning. In females, the sorting accuracy was not any better than from expected random chance frequency. Genotypes clustered more closely together in phenotypic space and the three‐group problem was separated with low accuracy. It may be possible to improve this prediction accuracy using different boundary selection protocol; however, it will not change the fact that the females are phenotypically more similar than males. The covariation of some of these phenotypic differences is still unclear, however, it may be possible to use these phenotypic associations in combination to predict genotypes of wild caught individuals. It will be our future task to investigate whether increasing data over the years and developing methods (e.g. convolutional neural networks) will enhance the prediction accuracy.

From an evolutionary standpoint, it is plausible that interplay between natural and sexual selection facilitates polymorphism in this species (Gordon et al., 2015, 2018; Nokelainen et al., 2012; Rönkä et al., 2020). Since male wood tiger moths, which are actively searching for females in the vegetation, have limited ability to see differences in yellow‐orange‐red hues (Henze et al., 2018), it is unlikely that sexual selection alone would be responsible for the colouration of females, but we cannot exclude the possibility that male colouration could be used in intraspecific communication. Moreover, recent studies in other species have shown that UV may facilitate separation of incipient species as recently demonstrated in *Colias* butterflies (Ficarrotta et al., 2022) and that the differences in UV reflection may arise from novel duplication of the gene producing sex‐specific differences in reflectance as in *Zerene cesonia* butterfly (Rodriguez‐Caro et al., 2021). Previous experiments have shown that birds learn to avoid red wood tiger moths more effectively than yellow or white ones (Ham et al., 2006; Lindstedt et al., 2011; Rönkä et al., 2018), but the selection for visual signals may be altered due to multimodal signalling (Rojas et al., 2018; Winters et al., 2021). Also, avian predators may distinguish between the nuances in colouration among the genotypes as they, and wood tiger moths, perceive UV wavelengths that are beyond human perception (Henze et al., 2018). Thus, ecologically relevant receivers, predators and conspecifics, may exert different selection pressures on visual signals beyond our perception (Endler, 1978) and maintain colour polymorphism in natural conditions (Galarza et al., 2014; Mochida, 2011; Nokelainen et al., 2014; Rönkä et al., 2020).

Conclusively, determining genotypes based on their phenotypic characteristics is important in any species, because it allows studying allele dynamics in the wild (e.g. as in tiger moths (Brakefield & Liebert, 1985; Fisher & Ford, 1947; Liebert & Brakefield, 1990), lizards (Sinervo & Calsbeek, 2006; Sinervo & Lively, 1996) and damselflies (Le Rouzic et al., 2015; Svensson & Abbott, 2005)). Colour polymorphisms have several fitness consequences in the maintenance of genetic variation (Galarza et al., 2014; Gray & McKinnon, 2007; McKinnon & Pierotti, 2010). They can influence intraspecific variation in mating cues (Merrill et al., 2012; Nokelainen et al., 2012), fitness of colour morphs in different light environments due to increased predation risk (Nokelainen et al., 2014, 2021; Rojas et al., 2014) and divergence in thermoregulatory capabilities (Forsman, 2000; Hegna et al., 2013; Lindstedt et al., 2009). As it has become possible to model the conspicuousness of different genotypes to different receivers (Endler & Basolo, 1998; Hart, 2001a; Henze et al., 2018), we may soon be able to estimate how their appearance shapes the fate of allelic combinations using long‐term data sets (Le Rouzic et al., 2015; Svensson & Abbott, 2005). Ultimately, this will broaden our understanding of how genetic variation underlying phenotypic evolution is shaped in nature.

## CONFLICT OF INTEREST

Authors have no conflict of interest to declare.

## AUTHOR CONTRIBUTIONS

ON wrote the first draft of the manuscript, conducted the image analysis and analysed frequency data; JAG devised the crossing‐design and inheritance analyses; JK helped with genotype–phenotype frequencies analyses; KS reared tens of thousands wood tiger moths over consecutive years and JM contributed substantially to project management and manuscript editing.

### PEER REVIEW

The peer review history for this article is available at https://publons.com/publon/10.1111/jeb.13994.

## Supporting information

Supinfo

## Data Availability

The supporting data are archived in a public repository (https://jyx.jyu.fi/handle/123456789/79808): https://doi.org/10.17011/jyx/dataset/79808.
