# Supplementary material for: Genetic colour variation visible for predators and conspecifics is concealed from humans in a polymorphic moth
Source: J Evol Biol. 2022 Mar 3;35(3):467–78. doi: 10.1111/jeb.13994 (PMC9314616; doi:10.1111/jeb.13994)

Supplementary material for Nokelainen et al. 2022 Journal of Evolutionary Biology

Title: Genetic colour variation visible for predators and conspecifics is concealed from humans in a polymorphic moth

Authors: Ossi Nokelainen[1,2*], Juan A. Galarza[1,2*], Jimi Kirvesoja[1], Kaisa Suisto[1] & Johanna Mappes[2,1] (*these authors contributed equally to this work)

Addresses: [1] Department of Biological and Environmental Science, University of Jyväskylä, P.O. Box 35, 40014 University of Jyväskylä, Finland

[2] Organismal and Evolutionary Biology Research Program, Faculty of Biological and Environmental Sciences, University of Helsinki, FIN-00014 Helsinki University, Finland

Copyright: All authors listed and affiliated universities. Published by Wiley on behalf of the European Society for Evolutionary Biology (ESEB).

Licence: Creative Commons Attribution 4.0 International (CC-BY 4.0)

Contents: Page

Supplementary tables

Table S1 2

Table S2 3

Table S3 4

Supplementary figures

Figure S1 5

Figure S2 6

Figure S3 7

Figure S4 8

Figure S5 9

Table S1: Observed versus expected phenotype frequency results of wood tiger moth genotype crossings and hindwing colour in males. White dominance over yellow hindwing colour was assumed. Small Chi Square values support the one-locus two-allele assumption (i.e., non-significant p-values support the Mendelian hypothesis). Sire comes first in the crossing table (i.e., father genotype x mother genotype). The information for genotype designation was derived from the pedigree using the ratios of the offspring. In total, 15 generations were produced over the course of six-years.

		Observed		Expected		Test statistics	
Crosses	N	whites	yellows	whites	yellows	Chi Square	*p*
Males							
WW x WW	301	299	2	301	0	<0.01	0.97
WW x Wy	66	66	0	66	0	0	1.0
Wy x WW	251	251	0	251	0	0	1.0
Wy x Wy	1834	1391	443	1376	456	0.53	0.46
yy x WW	722	707	15	722	0	<0.01	0.97
WW x yy	175	174	1	175	0	<0.01	0.97
Wy x yy	2344	1179	1165	1172	1172	0.04	0.77
yy x Wy	2202	1108	1094	1101	1101	0.08	0.76
yy x yy	3107	35	3072	0	3107	<0.01	0.97

Table S2: Observed phenotype frequency results of wood tiger moth genotype crossings in females. ‘Observed phenotypes’ refers to 1 to 6 scale from yellow to red, whereby 1-2 are yellow, 3-4 orange and 5-6 red-winged females based on human subjective evaluation on hindwing colour. The information for genotype designation was derived from the pedigree using the ratios of the offspring.

Genotype crossings				Observed phenotypes			
		yellow		orange		red	
	N	1	2	3	4	5	6
Females							
WW x WW	282	0	1	76	124	73	8
WW x Wy	70	0	7	17	34	7	5
Wy x WW	192	0	4	33	113	38	4
Wy x Wy	1608	0	28	261	827	473	19
yy x WW	615	0	15	93	286	207	14
WW x yy	168	0	7	30	89	42	0
Wy x yy	2099	2	52	358	986	672	29
yy x Wy	1936	5	41	264	943	654	29
yy x yy	2765	0	30	426	1393	861	55

Table S3: Boruta algorithm feature extraction results on the phenotypic variable importance.

Importance scores	mean	median	min	max	hits
Males (1st axis – white vs yellow)					
HW UV	17.65	17.58	15.99	20.15	1
HW sw	14.22	14.24	12.88	15.68	1
HW pattern diversity	11.70	11.65	9.80	12.98	1
HW pattern contrast	10.23	10.31	8.49	12.33	1
HW lw	8.05	8.01	6.68	9.60	1
Males (2nd axis – WW vs Wy)					
TH UV	10.31	10.38	7.44	12.08	0.98
TH/AB	6.55	6.52	3.64	9.30	0.96
FW/AB	5.33	5.43	2.97	7.47	0.90
AB UV	5.31	5.41	2.77	8.09	0.90
FW pattern diversity	4.51	4.50	1.86	6.90	0.82
Females (1st axis – white homozygotes vs yellow allele bearers)					
TH UV	14.23	14.26	12.42	16.55	1
AB marking size	12.35	12.46	10.58	13.72	1
AB UV	11.37	11.41	9.68	12.94	1
FW UV	10.29	10.23	8.67	11.85	1
HW sw	7.41	7.42	5.33	9.27	0.98
Females (2nd axis – Wy vs yy)					
AB marking size	9.86	10.04	6.49	12.09	1
HW sw	7.72	8.02	4.03	10.05	0.95
HW mw	6.04	5.95	1.79	8.15	0.89
FW sw	5.87	6.03	2.16	8.29	0.84
HW lw	5.27	5.35	2.80	7.19	0.83

Figure S1: Boruta feature selection algorithm results on the most important variables to differentiate genotypes in males. The top row describes five most important variables along the first discriminant axis (separates whites from yellows) whereas the bottom row shows the five most important variables along the second axis (separates WW from Wy). FW represents forewing, HW hindwing, TH thorax and AB abdomen.

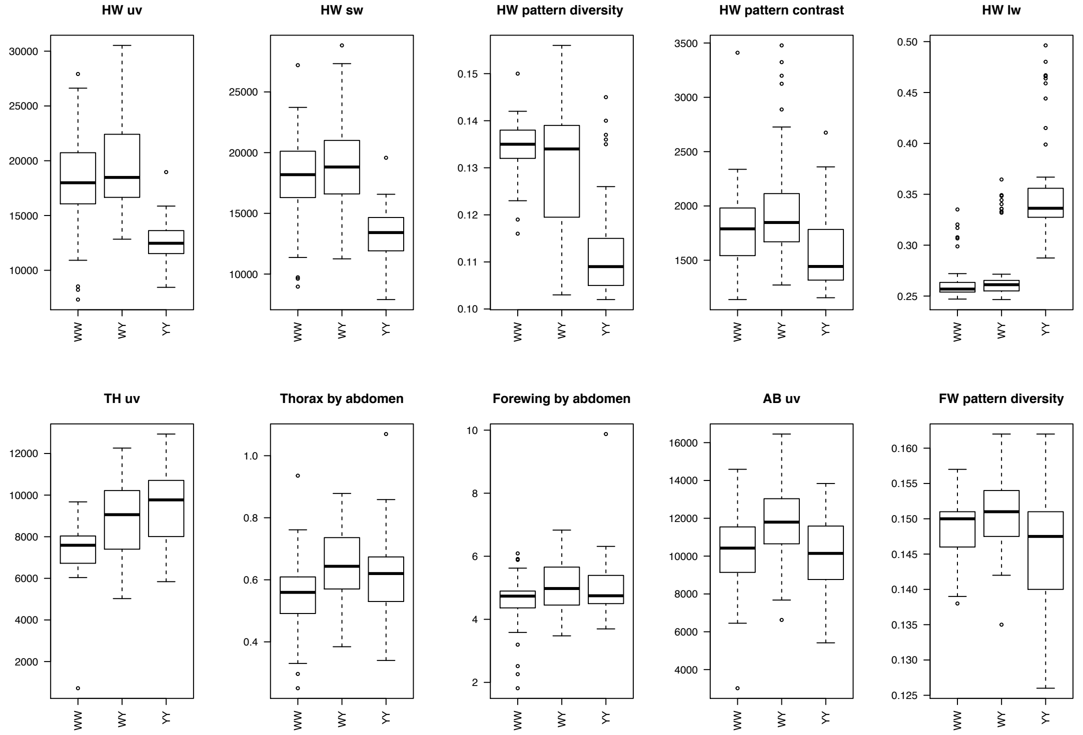

Figure S2: Boruta feature selection algorithm results on the most important variables to differentiate genotypes in females. The top row describes five most important variables along the first discriminant axis (separates white homozygotes WW from yellow allele bearers Wy, yy) whereas the bottom row shows the five most important variables along the second axis (separates Wy form yy). FW represents forewing, HW hindwing, TH thorax and AB abdomen.

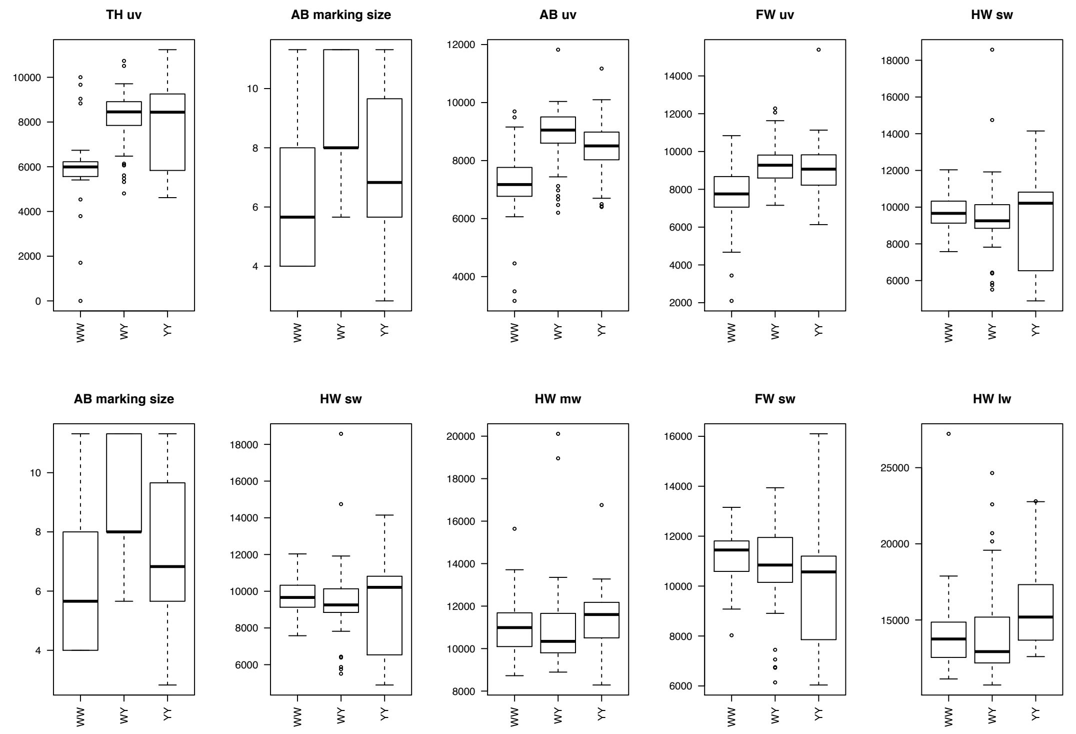

Figure S3: Wood tiger moth quiz – Part 1: Sequential sorting task example.

A) # - Tiger moth genotype quiz -

There are three genotypes (WW, WY, YY) both in males and females in Arctia plantaginis. Each one of these genotypes have subtle, but consistent differences in their appearance. Help us to understand how hard it is to classify their genotypes based on their looks.

B)

Male genotypes

Pay attention to general appearance, wing and body coloration. On average:

WW: White hind wings, disruptive forewing patterning, pale abdomen.

WY: White hind wings, yellow tinge in forewings and abdomen.

YY: Yellow hind wings, variable patterning, yellow abdominal colour.

Use these tips to classify the genotypes - the set starts on the next page.

C) Which genotype is this? M2 1 piste

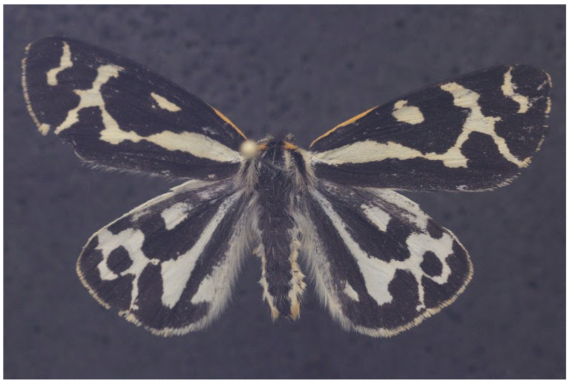

D)

Female genotypes

Pay attention to general appearance, wing and body coloration. On average:

WW: Red-orange hind wings, large and pale forewings and large, red abdomen.

WY: Reddest hind wings, large markings and red-orange tinge in abdomen.

YY: Yellowest hind wings, yellow tinge in abdomen, small in size.

Use these tips to classify the genotypes - the set starts on the next page.

E) Which genotype is this? F23 1 piste

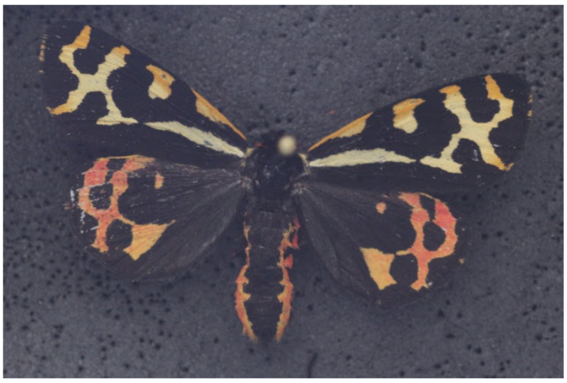

Figure S4: Wood tiger moth quiz – Part II: Simultaneous sorting task example.

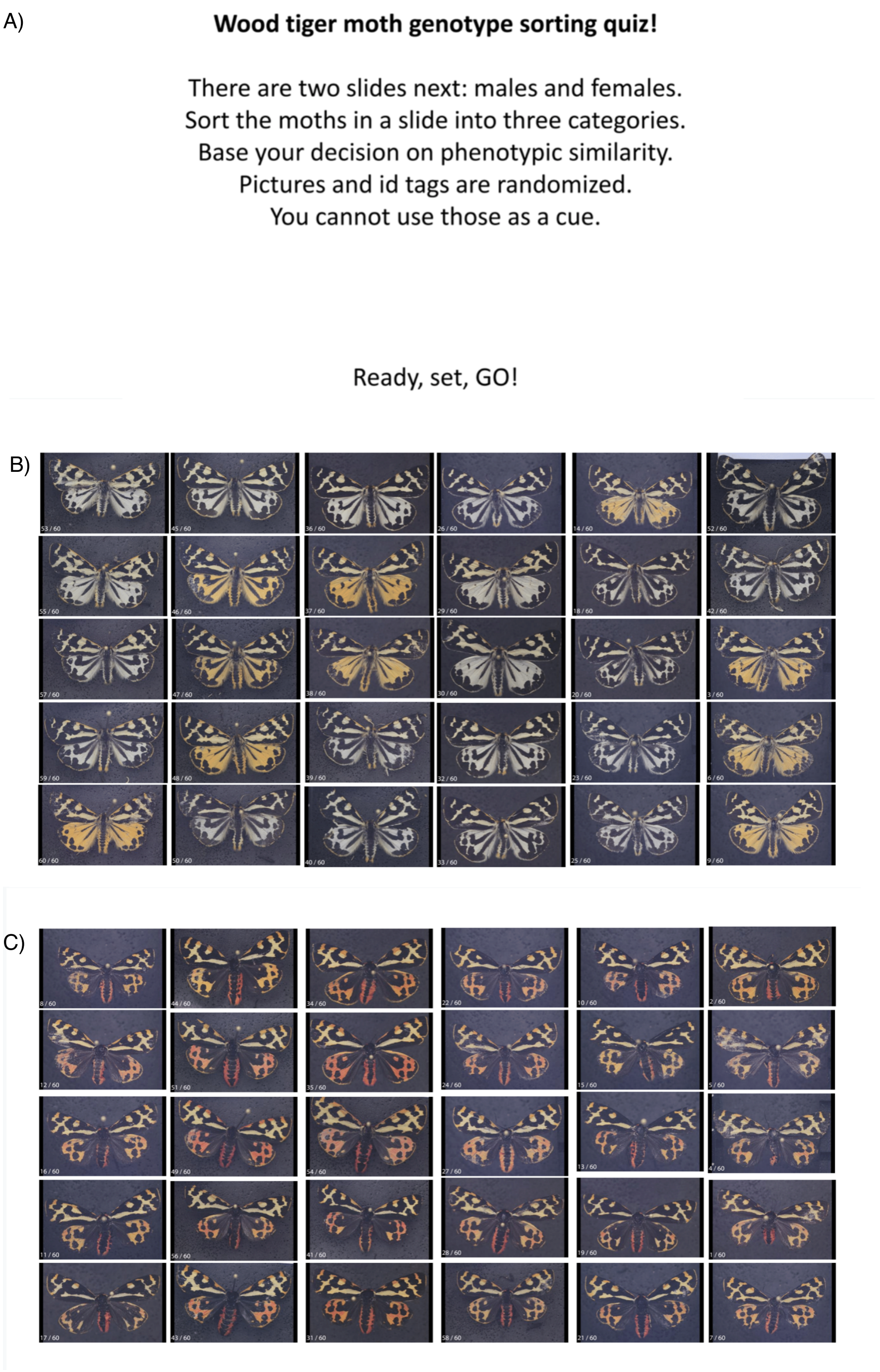

Figure S5: The objective versus subjective sorting of genotypes. Colour metric supervised computer algorithm (lda, linear discriminant analysis) outperforms human sorting accuracy of genotypes in both sequential ('sequence') and simultaneous ('sorting') tasks. In sequential sorting people were asked to classify moth pictures by their genotypes; only basic information of the best describing colour metrics were given. In simultaneous sorting, people were asked to sort the genotypes into three clusters based on their superficial appearance; no further instructions were given, but all moths of a sex were visible at the same time. Top panel row show results for the males, bottom for the females. Y-axis indicates the correct answers by genotype.

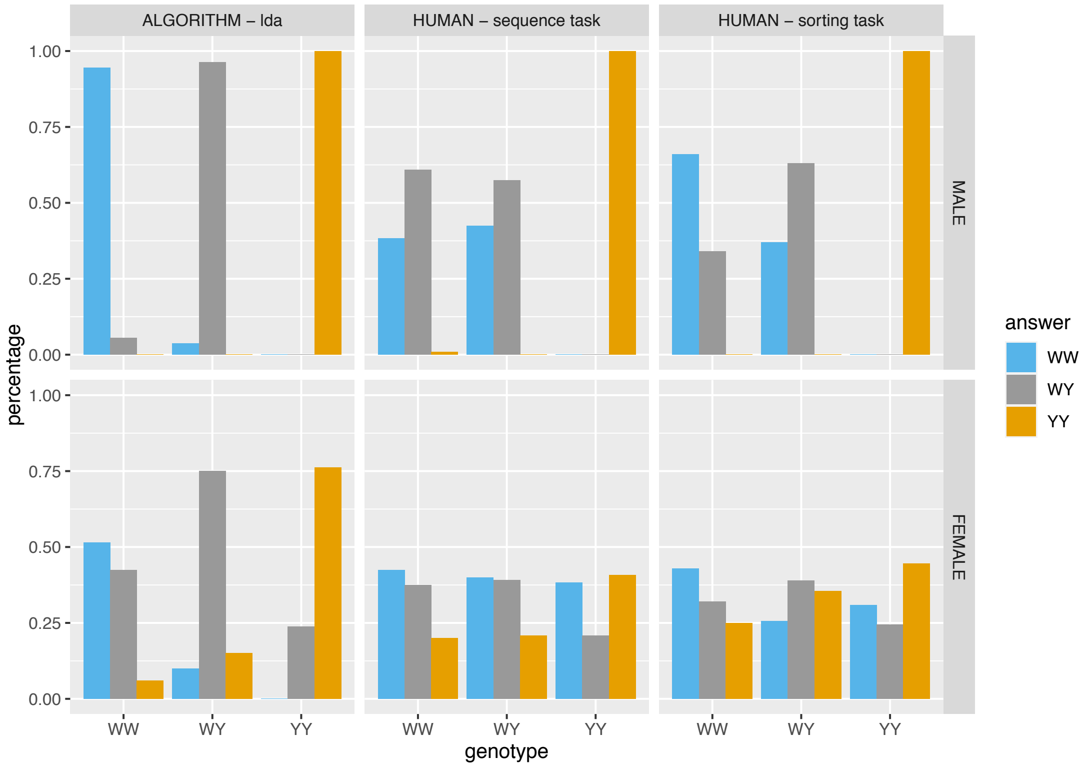

Supplement: Supplementary file 1 — Supinfo [file JEB-35-467-s001.pdf]
